# *Ulmus minor* response to Dutch elm disease: ***de novo*** transcriptome assembly and annotation

**DOI:** 10.1038/s41597-025-05539-y

**Published:** 2025-07-23

**Authors:** V. Chano, J. Sobrino-Plata, C. Martínez-Arias, C. Collada, J. Rodríguez-Calcerrada, J. A. Martín

**Affiliations:** 1 Departamento de Sistemas y Recursos Naturales, ETSI Montes, Forestal y del Medio Natural, Universidad Politécnica de Madrid. José Antonio Novais 10, 28040 Madrid, Spain; 2 Department of Forest Genetics and Forest Tree Breeding, Georg-August-Universität Göttingen. Büsgenweg 2, 37077 Göttingen, Germany; 3 Departamento de Genética, Fisiología y Microbiología, Facultad de Ciencias Biológicas, Universidad Complutense de Madrid. José Antonio Novais 12, 28040 Madrid, Spain; 4 Departamento de Biotecnología-Biología Vegetal, ETSI Agronómica, Alimentaria y de Biosistemas, Universidad Politécnica de Madrid, Avda. Puerta Hierro 2-4, 28040 Madrid, Spain; 5 Centro de I + D + i para la Conservación de la Biodiversidad y el Desarrollo Sostenible (CBDS), Universidad Politécnica de Madrid. José Antonio Novais 10, 28040 Madrid, Spain

**Keywords:** Agricultural genetics, Environmental sciences

## Abstract

Dutch elm disease (DED), caused by *Ophiostoma novo-ulmi* (ONU), has devastated elm populations across Europe and North America since the 20^th^ century. In this work, a *de novo* transcriptome assembly of *Ulmus minor* in response to ONU is presented. We used two DED-resistant genotypes, MDV2.3 and VAD2, and one DED-susceptible genotype, MDV1, to capture responses to ONU at four time points post-inoculation (6, 24, 72, and 144 hours). RNA from collected samples was isolated and sequenced producing 60.88 M 100 bp paired-end reads per sample. We performed a *de novo* transcriptome assembly combining data from the three genotypes. The assembly was functionally annotated and validated through differential gene expression analysis of the response. This dataset provides a valuable resource for studying molecular mechanisms of DED resistance in elms, contributing to broadening our understanding of tree immunity and facilitating potential applications in functional annotation of future genome assemblies.

## Background

The molecular defensive mechanisms and plant immune system have been widely studied in the plant model species *Arabidopsis thaliana*, together with other crop model species of great agronomic importance such as *Vitis vinifera*, *Oryza sativa* or *Solanum* sp.^1,2^. However, the scarcity of model species in forestry has hampered research on tree immune responses, while new climate conditions, together with the emergence of invasive pests and diseases, have evidenced the importance of forest health. Currently, improved and more cost-effective sequencing methods have enabled advances in tree breeding through genomic-assisted selection strategies, where a deeper understanding of the role of candidate genes in defensive pathways is crucial^3^. Recent efforts in transcriptomics have tried to close this gap providing evidence of tree immunity in both gymnosperms^4–7^ and angiosperms^8–12^.

One of the first reported cases of invasive alien diseases in forest species was the Dutch elm disease (DED), which has devastated elm populations in Europe and North America^13^. DED was initially caused by the vascular pathogen *Ophiostoma ulmi* during the first half of the 20^th^ century, later replaced by the more aggressive species *Ophiostoma novo-ulmi* (ONU), which is still very aggressive on the remaining elm populations. The pathogen is effectively transmitted by elm bark beetles of the genus *Scolytus* and *Hylurgopinus* during maturation feeding^14^. *Ulmus minor* in Europe and *Ulmus americana* in North America have been the most severely affected elm species, as nearly 100% of the populations of these two species are highly susceptible to ONU^13,15,16^.

Increasing the resistance to DED has been one of the primary objectives of different breeding programs^17^. This has been achieved by crossbreeding European elms with the more resistant Asian elm species, or through the selection and crossing of resistant native elms. This last strategy is usually preferred by environmental authorities to protect autochthonous genetic resources in reforestations^18^. The Spanish elm breeding program selected five native DED-resistant *U. minor* genotypes, which were registered as forest reproductive material^19^. These registered cultivars have opened new prospects to decipher and understand the molecular basis of DED resistance and elm immunity, which are crucial to restore elm forests. Transcriptomics and differential gene expression analysis have already been conducted on DED research. For instance, Perdiguero *et al*.^20^ provided a DED-sensitive reference transcriptome, with 58,429 unigenes (*de novo* assembly) from almost 1 M of high-quality reads and the identification of 22,500 single nucleotide polymorphisms (SNPs) among three *U. minor* genotypes (one susceptible and two resistant). This genetic resource was used for the design of a cDNA-microarray for later performing a time-course gene expression analysis of the DED-susceptible elm cultivar ‘Atinia’ (*U. minor* var. *vulgaris*^21^), in response to ONU^22^. More recently, Islam *et al*.^10^ described the transcriptomic responses of a susceptible and a resistant genotype of *U. americana* to ONU, providing 5,329 Differentially Expressed Genes (DEGs) in response to the infection. In this work, we aimed to create a reference transcriptome for elm through a time-course design that captures the local and systemic responses at four time points after *U. minor* inoculation with ONU. To this end, we used two resistant Spanish genotypes, MDV2.3 and VAD2, which show differences in their response to the disease^23^, along with the susceptible Spanish genotype MDV1.

## Material and Methods

### Plant material and experimental design

Three *U. minor* genotypes were used in this work. Two of them were registered in 2014 as Spanish forest reproductive material under the names “Dehesa de Amaniel” (hereinafter referred to as “MDV2.3”, from Dehesa de la Villa, Madrid, Spain) and “Ademuz” (VAD2 hereinafter, from Ademuz, Valencia, Spain). MDV2.3 and VAD2 are highly resistant to DED^19^. The third *U. minor* genotype used was MDV1 (from Dehesa de la Villa, Madrid, Spain), which is highly susceptible to the disease^19^. Buds from the three genotypes were used as starting material for *in vitro* micropropagation in growing chambers according to Conde *et al*.^24^. One-month-old plantlets were transplanted to 500 mL pots filled with peat (Kekkilä®, Vantaa, Finland) and perlite in volumetric proportions 3:1 as substrate. The pots were placed in a growth chamber with 16 h photoperiod using fluorescent white light with approximately 240 µmol m-^2^ s-^1^ photosynthetically active radiation at 24/18 °C day/night temperature and 60% air relative humidity. Plants were regularly watered with filtrated tap water for one month.

In planta inoculations were performed on 2-month-old plants using a ONU bud-cell water suspension at a concentration of 10^7^ blastospores ml^-1^. Pathogen spores were delivered into the xylem sap stream through a transverse cut made with a sharp blade in the base of the stem^25^. To obtain the ONU blastospores, mycelial plugs from the *O. novo-ulmi* ssp. *americana* isolate Z-BU1 were grown in Erlenmeyer flasks containing Tchernoff’s liquid medium^26^ at 22 °C in the dark under constant shaking to induce sporulation. Three days later, blastospores were collected by centrifugation and the concentration adjusted to 10^7^ blastospores ml^-1^ using a haemocytometer. A total of 32 plants from each genotype were used, half of them inoculated with 10 µl of the bud-cell suspension *O. novo-ulmi* ssp. *americana* isolate Z-BU1^17^, and the other half mock-inoculated with 10 µl of sterilized distilled water. Samples were collected following a time series with four sampling points: 6 hours post inoculation (hpi hereinafter), 24 hpi, 72 hpi and 144 hpi. Four clonal replicates (ramets) were used as biological replicates for each experimental group. Additionally, two stem segments were collected per plant at each sampling time: one 4-cm-long segment including the inoculation point (local response) and a similar segment taken 10 cm above the inoculation point (systemic response). Plant material was immediately frozen in liquid nitrogen after collection, grinded into a fine powder using a Retsch® mill and sterilized stainless steel balls and stored at −80 °C until use.

### RNA isolation and sequencing

Total RNA was extracted from the 192 samples using a Plant/Fungi total RNA Purification Kit (Norgen Biotek Corp., Thorold, Ontario), following the instructions from the manufacturer. The protocol included a DNase treatment step for removal of residual DNA with the Norgen RNase-free DNase I Kit (Norgen Biotek Corp., Thorold, Ontario). The integrity of isolated RNA was assessed using a 2100 Bioanalyzer (Agilent Technologies). Approximately 40 µl of total RNA per sample were submitted to Macrogen (Macrogen, The Netherlands), and 192 TruSeq mRNA stranded libraries were prepared using polyA selection. One hundred bp paired-end reads were generated with Illumina HiSeq 3000, with a sequencing depth of 50 million reads per sample. Samples were multiplexed in 4 lanes with 6 samples per lane. In order to avoid technical biases due to sample position in the sequence, biological replicates for each sample were randomly distributed among lanes. Reads were demultiplexed based on barcodes included during library preparation.

### Bioinformatic processing of sequencing data

The bioinformatic pipelines were run on a Linux Ubuntu v18.04 workstation featuring an Intel Xeon Gold 6,130 2.1 GHz processor with 32 cores (64 threads) and 256 GB RAM. Storage included a 2TB SSD (35200 MBps reading and 2500 MBps writing speed) and a Redundant Array of Inexpensive Disks (RAID) setup with 4 TB HDDs (7200 rpm, 12 Gb/s). The home system (SSD) hosted the software, while datasets and databases were stored in the RAID.

Initial quality control of raw reads was performed by using FastQC v0.11.7 and MultiQC^27^. We then used Trimmomatic 0.36^28^ with a Phred Score below 30, excluding reads with a minimal length of 30 nucleotides, and using a sliding window (nucleotide window size) of 4. We also cut 12 nucleotides from the beginning of reads using the headcrop option and removed possible adapter sequences when present. Finally, the improvement of quality of trimmed and filtered reads was again assessed with FastQC and MultiQC. By using HISAT2^29^, processed fastq files were mapped against the genome available for the ONU reference strain H327^30^ (downloaded from the DOE Joint Genome Institute, https://mycocosm.jgi.doe.gov/Ophnu1/Ophnu1.home.html) to filter out reads from the pathogen. First, ONU genome was indexed using the routine hisat2-build, and mapping was performed with the option –score-min L,0,-0.6. Mapped reads were saved as bam files and stored for further analysis (data not shown). Unaligned reads were saved as fastq files and mapped against a custom database created ad hoc and including sequences from bacteria, mostly riRNA downloaded from SILVA (https://www.arb-silva.de/), and from endophytes putatively present in plant samples from the FungDB (https://fungidb.org/fungidb/app). Those sequences that did not map during any of these mapping steps were considered as elm reads and used for *de novo* transcriptome assembly and validation analyses.

### ***De novo*** transcriptome assembly and functional annotation

Reads were *de novo* assembled using Trinity v2.14.0^31^, indicating a kmer size of 32 for the de Bruijn graph construction, which was selected after running Kmergenie v1.7051^32^. Due to the high number of initial reads and, consequently, the significant computational requirements, assembly was first performed individually for each genotype (MDV1, MDV2.3 or VAD2). Afterwards, a single transcriptome was meta-assembled with these three genotype-transcriptomes together with reads from a 454-pyrosequencing conducted in a previous work^19^, and corresponding to the moderately resistant genotypes MDV4.5 (from Dehesa de la Villa, Madrid, Spain) and JCA2 (from Cazorla, Jaén, Spain), and the susceptible TOAL1 (from Algodor, Toledo, Spain). Unlike Illumina sequencing, which results in numerous short-reads, 454-pyrosequencing allows obtaining longer reads that may be useful to fill possible gaps and reduce redundancies. The sra file from this 454-pyrosequencing was first downloaded from NCBI Short Read Archive, accession number SRR1687227, and then converted into fastq by using prefetch and fastq-dump routines, respectively, from SRA-Toolkit v2.10.2 (https://trace.ncbi.nlm.nih.gov/Traces/sra/sra.cgi?view=software). This fastq file was also processed with Trimmomatic and evaluated with FastQC, as previously indicated for Illumina reads. Compared to individual transcriptomes of the three genotypes, the number of transcripts was highly reduced in the single transcriptome, although it still contained many redundancies as isoforms. Therefore, we included a final step to identify so-called unigenes and generate a single reference transcriptome by using the perl-based tool get_longest_isoform_seq_per_trinity_gene.pl. Contigs included in the final transcriptome assembly were first renamed following the alphabetical order given by Trinity.

Transcripts were annotated using a multi-fasta file as input for local version of BLASTx^33^ using TOA v0.66^34^ and the RefSeq Viridaplantae section. Parameters selected for BLASTx were an e-value threshold of 10^-5^ and a maximum of 50 hits per query (transcript). The output files (xml format) were then uploaded into the Functional Analysis module (formerly known as Blast2GO) from Omicsbox v4.0^35^. Mapping and Annotation tools from Omicsbox were used for the assignment of Gene Ontology (GO) terms associated to these transcripts, and InterPro scan was also used to improve annotation, by retrieving GOs associated with protein motifs/domains. The GO-Slim tool was then used to filter and reduce these GO terms to those associated with plants. Moreover, information about Enzyme Codes (EC) and pathways from the Kyoto Encyclopaedia of Genes and Genomes (KEGG) was also gathered by means of Omicsbox.

### Counting and differential gene expression analyses for transcriptome validation

Local samples (i.e. taken at the inoculation point) of ONU-inoculated and mock-inoculated plants from the three genotypes were used for the validation of the transcriptome assembly. The pseudo-aligner Kallisto v0.46^36^ was used to index the reference transcriptome of *U. minor* and generate raw counts for all the transcripts per sample library, creating a final count matrix later imported in R for differential gene expression analysis using DESeq2 v1.30^37^. Prior to differential gene expression analysis, counts were normalized using the vst (variance stabilizing transformation) function, and exploratory analyses of normalized gene expression values were carried out by Principal Component Analysis (PCA) and sample-to-sample distances implemented in DESeq2.

Differential gene expression analyses were performed for each genotype and single time point by using Wald’s test to compare ONU-inoculated and mock-inoculated samples (including four biological replicates each). A logarithmic Fold Change (LFC hereinafter) value was obtained for each transcript, calculated as the logarithm of the expression ratio between ONU-inoculated and mock-inoculated samples. Therefore, the LFC value for a given transcript resulted in a positive number when the expression was higher in ONU-inoculated plants (referred hereinafter as overexpression, up-regulation or induction) whereas a negative number (referred hereinafter as underexpression, down-regulation or repression) indicated a higher expression in mock-inoculated plants. The model formula for the Wald’s test in DESeq2 was indicated as “design = ~ treatment”, and differentially expressed genes (DEGs) were selected when the p-value (adjusted for multiple comparisons by False Discovery Rate, FDR^38^) was lower than 0.05 and the LFC greater than the absolute value of 2 (adj p-value < 0.05 and LFC > |2|). Resulting time-specific sets of DEGs were compared among genotypes by creating an upset plot with the R package upsetR v1.4.0^39^.

## Data Records

This Transcriptome Shotgun Assembly project has been deposited at DDBJ/EMBL/GenBank under the accession GLCK00000000^40^. The version described in this paper is the first version (GLCK00000000.1). Supplementary Tables and multi-fasta file with transcriptome assembly were deposited in Figshare under a CC-By license^41^. Raw sequencing data (fastq files) of local and distal samples of *U. minor*, ONU-inoculated and mock-inoculated along a time-course (6-, 24-, 72- and 144 hpi) were deposited at the National Center for Biotechnology Information (NCBI) under the BioProject accession no. PRJNA1226172, and the Sequence Read Archive (SRA) accession no. SRP566393^42^.

## Technical Validation

**Fig. 1:**
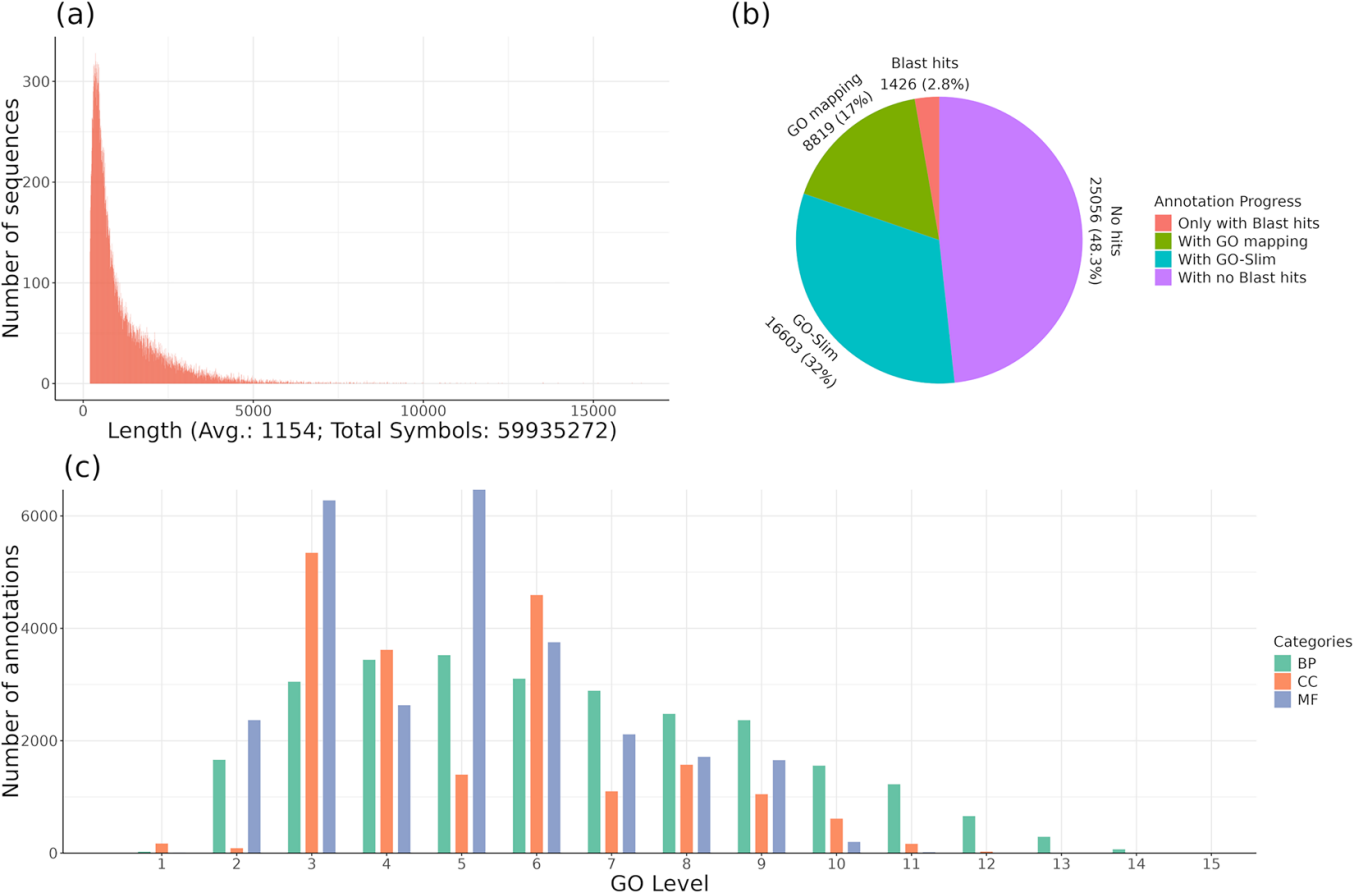
Final transcriptome assembly. (**a**) Size distribution of unigenes after generating a single reference transcriptome. (**b**) Functional annotation progress (**c**) Distribution of Gene Ontology (GO) levels for the three categories Biological Process (BP), Cellular Component (CC) and Molecular Function (MF).

In this work, we performed a *de novo* transcriptome assembly of the *U. minor* response to DED. Based on the initial 11,689.4 M paired-end reads (60.88 M paired-end reads per sample), a first transcriptome pre-assembly for each genotype was performed. These pre-assemblies yielded 228,642 contigs for the DED-susceptible MDV1, 233,398 contigs for the DED-resistant MDV2.3, and 242,861 contigs for the DED-resistant VAD2. The three *de novo* primary transcriptomes were then meta-assembled together with 971,002 reads (350 bp length) derived from a 454-pyrosequencing previously performed for *U. minor*^20^ into a preliminary single transcriptome assembly, resulting in 74,593 contigs (including isoforms). The resulting quality parameters were: 54,995 contigs over 500 bp and 33,658 over 1,000 bp, 96 464,235 bp total length, 1,293.21 and 881 bp average and median contig length, respectively, 38.74% GC content, and N50 and L50 values of 1,961 bp and 15,439, respectively (Table 1). The longer transcripts per isoform were identified, reducing redundancies and obtaining a final transcriptome assembly that included 51,507 unigenes^40^. Assembly quality parameters were: 35,324 and 19,495 contigs over 500 and 1,000 bp, respectively, 59 715,254 bp total length, 1,159.36 and 733 bp average and median contig length, respectively, 38.27% GC content, and N50 and L50 values of 1,810 bp and 9,996, respectively (Table 1). Top5 contigs with maximum length were Trinity_DN1091_c0_g1_i4 (16,407 bp), Trinity_DN9258_c0_g1_i2 (16,140 bp), Trinity_DN1582_c0_g1_i1 (15,135 bp), Trinity_DN1263_c0_g2_i1 (14,705 bp) and Trinity_DN25886_c0_g1_i1 (13,962 bp). The distribution of the number of sequences by sequence length is shown in Fig. 1a. In Perdiguero *et al*.^20^, the previous transcriptome resulted in 58,429 unigenes, a slightly higher but very similar number compared to the current work. Similar numbers were also obtained in a more recent study on DED affecting *U. americana*^10^, with 55,499 unigenes identified.

**Table 1:** Summary of transcriptome assembly quality parameters.

Parameters	Preliminary assembly	Final assembly
No. of contigs	74,593	51,507
No. contigs >500 bp	54,995	35,324
No. contigs >1000 bp	33,658	19,495
Total lenght (bp)	96 464,235	59 715,254
Largest contig (bp)	16,407	16,407
Average contig length (bp)	1,293.21	1,159.36
Median contig length (bp)	881	733
GC content (%)	38.74	38.27
N50 contig size (bp)	1,961	1,810
L50 (contig no.)	15,439	9,996

bp: base pairs; no.: number; GC: guanine-cytosine; N50: sequence length of the shortest contig at 50% of total assembly length; L50: count of smallest number of contigs with sum length over 50% of total assembly length.

### Functional annotation of *Ulmus minor* transcriptome

The functional annotation of the final list of unigenes^40^ is presented in Supplementary Table S1. From the initial 51,507 sequences, 26,566 (51.6%) matched to protein records in RefSeq Viridiplantae (Fig. 1b). From these, 1,341 sequences (2.6%) did not yield GO terms associated, while 8,768 sequences (17%) were associated with GOs and 16,457 (32%) passed the GO-Slim step. The percentage of matched sequences was higher than the 42% obtained for *U. americana*^10^. In addition, 24,461 sequences were matched after InterPro Scan, retrieving GOs from 12,473 of them. Thus, the absolute number of identified GO terms was 73,799 distributed throughout three categories, Cellular Component (CC), Molecular Function (MF), and Biological Process (BP), classified up to 15 levels of specificity (Fig. 1c). A direct count within these three categories (data not shown) revealed that the top-five GO terms where: CC) “membrane”, “nucleus”, “cytoplasm”, “plasma membrane” and “chloroplast”; MF) “protein binding”, “nucleotide binding”, “hydrolase activity”, “catalytic activity” and “transferase activity”; and BP) “biosynthetic process”, “nucleobase containing compound metabolic process”, “cellular protein modification process”, “transport” and “cellular process”.

### Differential gene expression analysis: ONU-inoculated vs. mock-inoculated samples

**Fig. 2:**
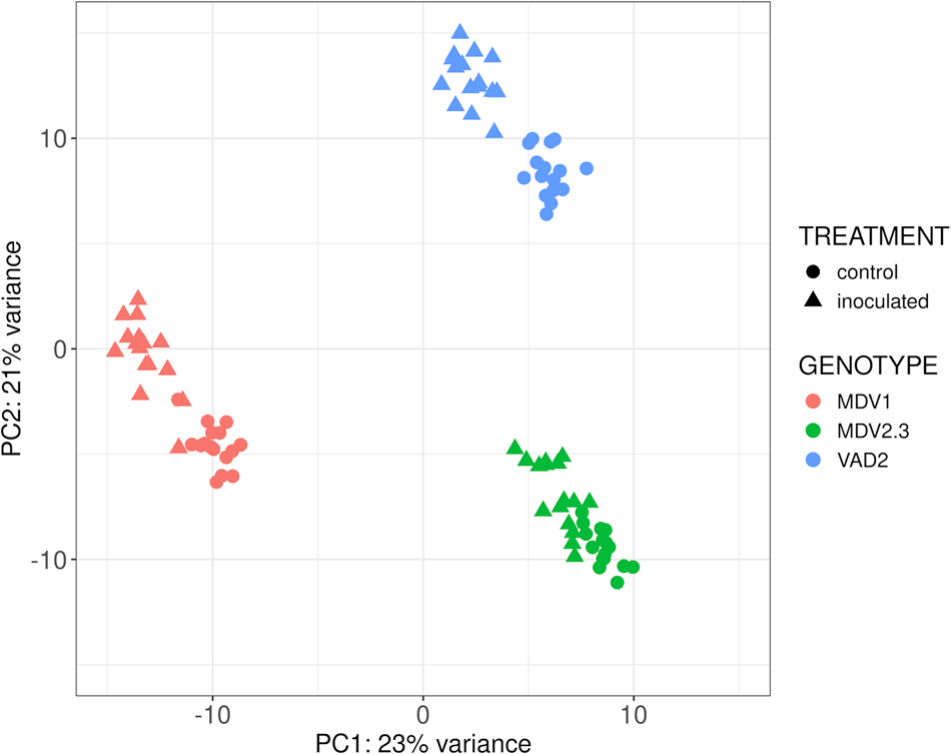
Principal Component Analysis (PCA) of transcriptomic local profiles from three *Ulmus minor* genotypes (MDV1, MDV2.3, and VAD2), following inoculation with *Ophiostoma novo-ulmi* or mock-inoculation with sterile water (control). Expression values were normalized by vst (variance stabilizing transformation).

The gene expression matrix was imported in R and total count values were normalized by means of the *vst* (variance stabilizing transformation) method. Normalized expression values were then inspected through a PCA (Fig. 2). The three genotypes were clearly separated by the first component (PC1; 23% of the variance), with a greater variance between the DED-susceptible MDV1 and the two DED-resistant genotypes MDV2.3 and VAD2. Interestingly, the second component (PC2, 21% of the variance) separated the susceptible MDV1 and the resistant MDV2.3, both from the same origin (Dehesa de la Villa, Madrid), from the resistant VAD2, with a different origin (Ademuz, Valencia). In addition, the two sub-groups corresponding to the inoculation treatment (ONU-inoculated and mock-inoculated samples) were distinguished within each genotype. For the DED-susceptible MDV1 (Supplementary Fig. S1a), ONU-inoculated and mock-inoculated trees were separated by the first component (29% of the variance), while the second component separated the different collecting-points across the time-course (24% of the variance). For the DED-resistant MDV2.3 (Supplementary Fig. S1b), the first component (22% of the variance) separated the time points, while the second component (13% of the variance) separated ONU-inoculated from mock-inoculated samples. Similarly to MDV1, ONU-inoculated and mock-inoculated VAD2 trees were also separated by the first component (24% of the variance), while separation of time points was observed in the second component (18%) (Supplementary Fig. S1c). These PCAs revealed distinct responses among genotypes. Additionally, the results demonstrated the high effectiveness of the inoculation method across all individuals, which also exhibited time-dependent responses. Similar responses were also observed in other tree diseases like ash dieback^11,12^, pine pitch canker in *Pinus pinaster* and *Pinus pinea*^5,7^, or needle bladder rust in Norway spruce^6^.

**Fig. 3:**
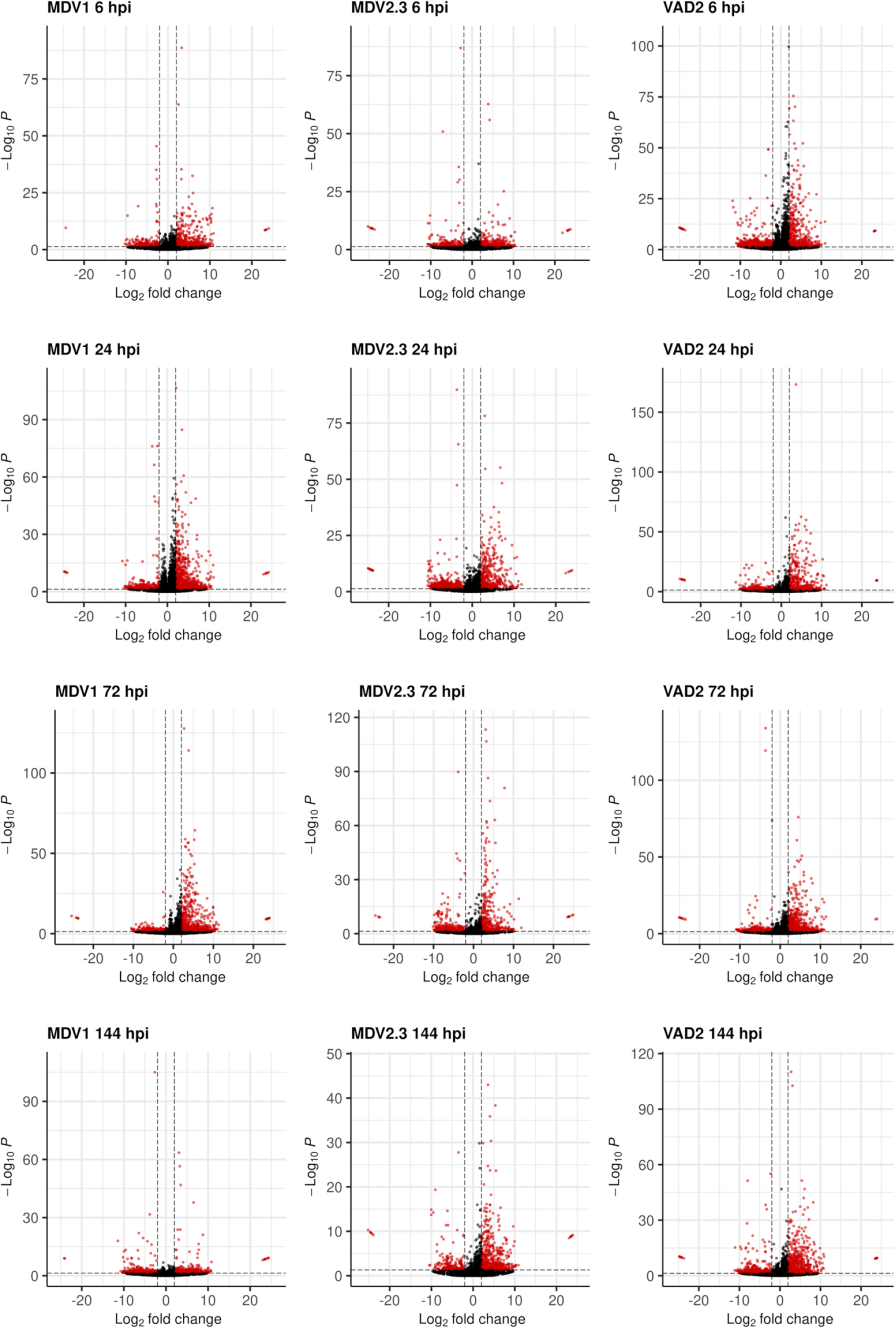
Genotype- and time-specific volcano plots showing differentially expressed genes (DEGs, red dots) between *Ophiostoma novo-ulmi*-inoculated and mock-inoculated samples across four time points (6-, 24-, 72-, and 144-hours post-inoculation, hpi) for each *Ulmus minor* genotype (MDV1, MDV2.3, and VAD2). Dashed lines represent significance thresholds (FDR-adjusted P < 0.05, horizontal) and expression cutoffs (log_2_ fold change ≥ |2|, vertical).

**Fig. 4:**
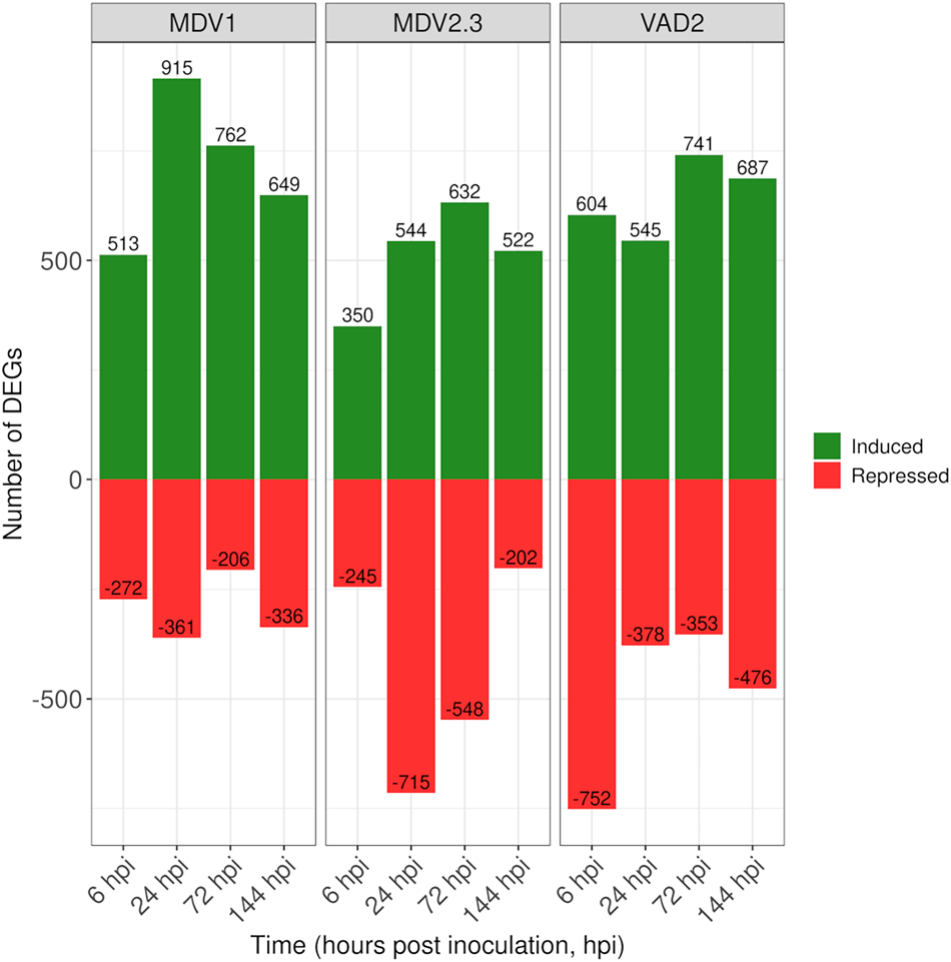
Bar plots displaying the number of differentially expressed genes (DEGs) per time point (6-, 24-, 72-, and 144-hours post-inoculation, hpi) and *Ulmus minor* genotype (MDV1, MDV2.3, and VAD2). For clarity, upregulated genes are shown as positive values, while downregulated genes are shown as negative values.

Validation of the *de novo* transcriptome was performed by using this final assembly as reference for the differential gene expression analysis of the local responses to DED for each genotype and sampling time across the time-course experiment. The distribution of DEGs for the three genotypes and four time points is depicted in Fig. 3. DEGs (red dots) were considered when adjusted p-values < 0.05 and LFC > |2|. Overall, the number of up-regulated and down-regulated genes in response to the infection differed between genotypes and time points, showing different profiles not only between DED-susceptible and DED-resistant genotypes but also between the two DED-resistant genotypes (Fig. 4). The number of induced transcripts in the susceptible genotype MDV1 increased from 6 to 24 hpi, later decreasing, while the number of repressed genes slightly varied during the response (Supplementary Tables S2-S5). Similarly, the number of induced genes in the resistant genotype MDV2.3 also increased during the response from 6 to 72 hpi, decreasing only at 144 hpi (Supplementary Tables S6–S9). In addition, the number of repressed transcripts increased from 6 to 24 hpi, later decreasing (for clarity, the number of down-regulated genes in Fig. 4 are represented as negative numbers). In contrast, the resistant VAD2 showed a different response, with more genes being induced at 6 hpi than at 24 hpi, later increased at 72 hpi and decreased at 144 hpi. Moreover, the profile of repressed genes during the time-course was also different than in MDV1 and MDV2.3, with the highest number of down-regulated genes found at 6 hpi, decreasing later and kept in similar values during the rest of the analysed response (Supplementary Tables S10–S13).

In summary, a first inducible local response to the infection was found for the three genotypes, characterized by a remarkably higher number of up-regulated than down-regulated genes, particularly in the susceptible genotype. The DED-susceptible MDV1 and the DED-resistant MDV2.3 showed a similar number of total DEGs involved in the local response (3,033 and 3,029, respectively) and also a similar response pattern, especially regarding the proportion of induced/repressed genes and their variation across the response. However, the number of total DEGs involved in the local response of the resistant VAD2 was higher (3,325 DEGs). It was also noteworthy that the two DED-resistant genotypes, MDV2.3 and VAD2, showed marked differences in their responses. The expression profile regarding the number of DEGs within a given sampling time was different for VAD2 compared to the other two genotypes, as the greatest transcriptomic response was produced at 24 hpi for MDV1 and MDV2.3, and at 6 hpi for VAD2.

**Fig. 5:**
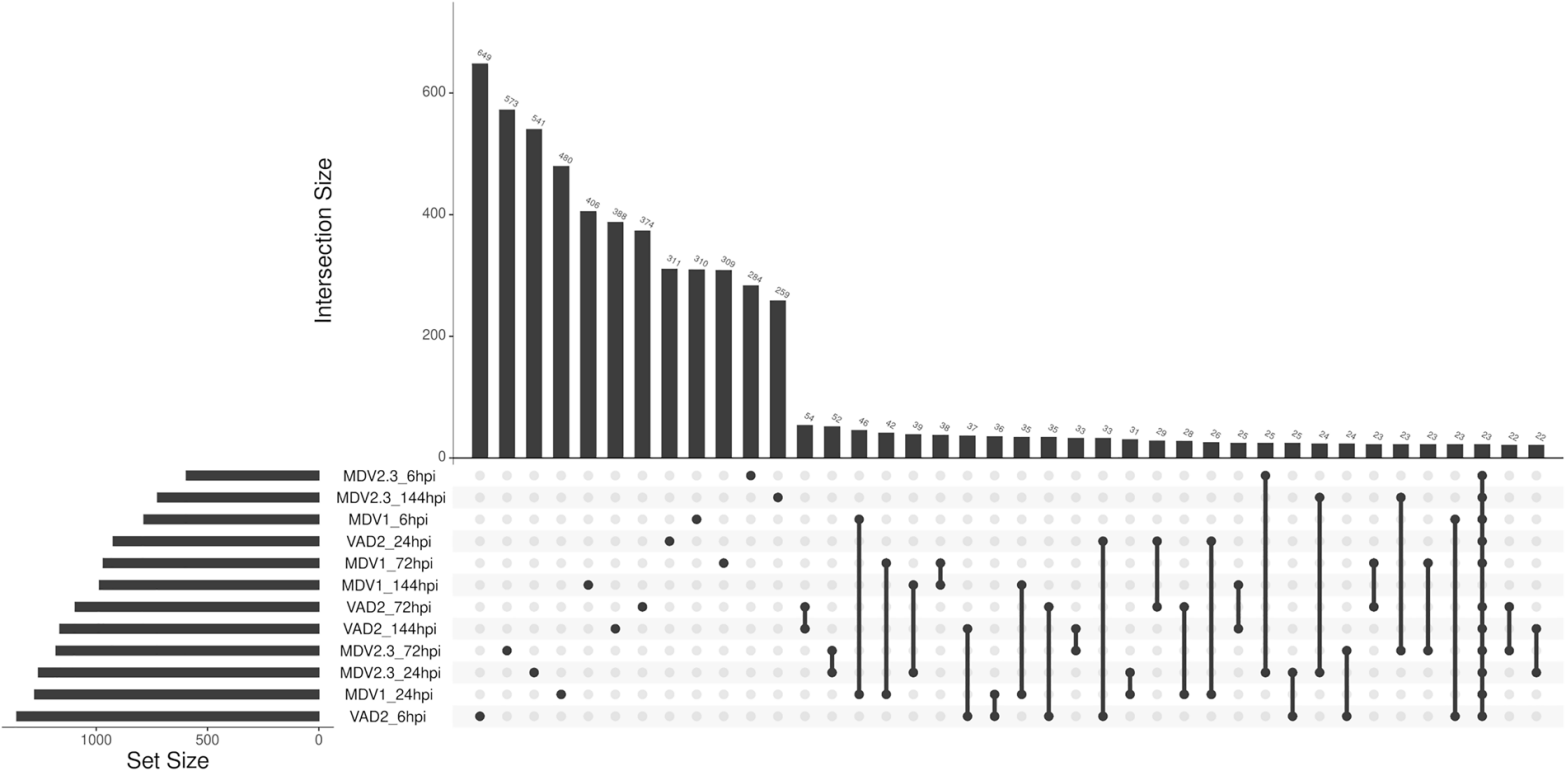
UpSet plot illustrating shared and exclusive differentially expressed genes (DEGs) across genotypes (MDV1, MDV2.3 and VAD2) and time points (6-, 24-, 72-, and 144-hours post-inoculation, hpi). For visualization purposes, the number of intersections is limited to a maximum of 40 DEG sets.

As shown in the top barplot in Fig. 5, intersections were sorted in the *x* axis by the sizes (number of DEGs) in *y* axis, indicating a considerably higher number of DEGs that were exclusively found under each time point and genotype, while the first groups of shared genes were found between two time-points within one genotype (Supplementary Table S14). These findings align with those observed in a longer-term time-course analysis (from 7 to 28 days post-inoculation) of ash dieback response, which identified a higher number of genotype- and time-specific DEGs compared to shared DEGs^11^. The scarcity of common responses, particularly in resistant genotypes, may be explained by a lack of close co-evolution between *U. minor* and DED pathogens, as evidenced by the high susceptibility and mortality of field elms during the two DED pandemics^43^.

## Usage Notes

This dataset is a collection of a transcriptome assembly^40^, functional annotation and expression profiles of the local response to DED in a time-course experiment (at 6-, 24-, 72- and 144 hpi) in three *U. minor* genotypes showing different levels of resistance to the disease. Further research can leverage these raw reads and the annotation file for the identification of genomic features and improve future genome assembly. In addition, researchers can use this assembly as a reference for further transcriptomic profiling of elms in response to DED. The catalogue of candidate genes proposed here, with special focus on genes involved in plant immunity and defensive mechanisms, opens a vast range of opportunities to further investigate the genetic basis of elm susceptibility/resistance to DED through complementary analyses.

## Supplementary information

Supplementary Figure S1

Supplementary Tables S1 - S14

## Data Availability

Bioinformatic pipelines and R scripts for this work were uploaded in GitHub (https://github.com/vchano/genesis_transcriptome.assembly).
