# Supplementary material for: Ulmus minor response to Dutch elm disease: de novo transcriptome assembly and annotation
Source: Sci Data. 2025 Jul 23;12:1282. doi: 10.1038/s41597-025-05539-y (PMC12287513; doi:10.1038/s41597-025-05539-y)
Supplement: Supplementary file 1 — Supplementary Figure S1 [file 41597_2025_5539_MOESM1_ESM.pdf]

scientific data

Scientific Data. Supplementary Material.

Ulmus minor response to Dutch elm disease: de novo transcriptome assembly and annotation

Chano V, Sobrino-Plata J, Martínez-Arias C, Collada C, Rodríguez-Calcerrada J, Martín JA

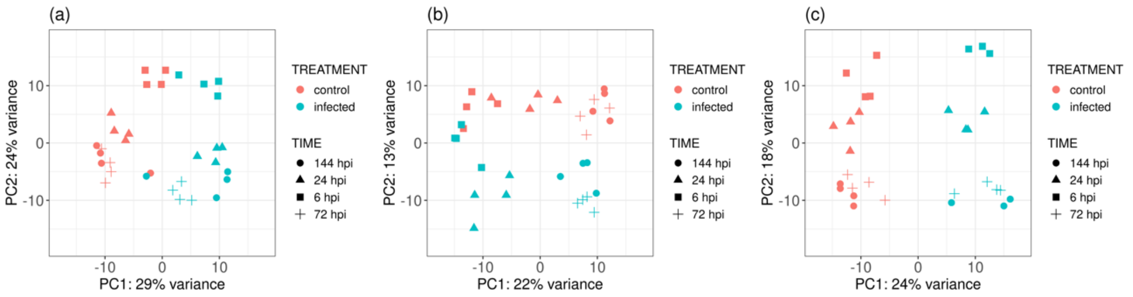

Supplementary Figure S1. Principal Component Analysis (PCA) of normalized gene expression values from three genoypes of *Ulmus minor* showing different responses to Dutch elm disease (DED). (a) PCA of normalized expression values of inoculated and mock-inoculated samples of DED-susceptible genotype MDV1 along a time-course analysis at 6-, 24-, 72- and 144 hours post inoculation (hpi). (b) PCA of normalized expression values of inoculated and mock-inoculated samples of DED-resistant genotype MDV2.3 along a time-course analysis at 6-, 24-, 72- and 144 hpi. (c) PCA of normalized expression values of inoculated and mock-inoculated samples of DED-resistant genotype VAD2 along a time-course analysis at 6-, 24-, 72- and 144 hpi.
